# Role of poly-herbo-mineral combination in management of *kunakha* (paronychia)

**DOI:** 10.1186/s13256-025-05588-2

**Published:** 2025-10-29

**Authors:** Harshada Timande, Mayuri Amol Deshpande, Amol Madhav Deshpande, Akshay Pargaonkar

**Affiliations:** 1 Wardha, Maharashtra India; 2 Department of Kayachikitsa, Mahatma Gandhi Ayurved College and Research Centre, Datta Meghe Institute of Higher Education and Research (Deemed to be University) Salod (H), Wardha, Maharashtra India; 3 Department of Rachana Sharir, Mahatma Gandhi Ayurved College and Research Centre, Datta Meghe Institute of Higher Education and Research (Deemed to be University) Salod (H), Wardha, Maharashtra India; 4 Department of Dravyaguna, Mahatma Gandhi Ayurved College and Research Centre, Datta Meghe Institute of Higher Education and Research (Deemed to be University) Salod (H), Wardha, Maharashtra India

**Keywords:** Kunakha, Paronychia, Nail bed infection

## Abstract

**Background:**

Paronychia, a bacterial or fungal infection affecting the area where the nail meets the skin, often impacts individuals engaged in frequent water-related work. Typically resolving within a week, the condition can persist and worsen with exposure to water, chemicals, or unclean substances, leading to chronic infection and significant cosmetic concerns. In Ayurveda, this condition is referred to as *Kunakha*, a term originating from “Ku” (bad) and “Nakha” (nail) in Hindi. *Kunakha*, associated with *Asthi dushti* (Bone tissue) and characterized by nail discoloration, pain, and chronicity, corresponds to Kshudraroga in Ayurvedic texts, particularly the 13th chapter of Nidana Sthana by Acharya Susruta. The condition results from vitiated Tridosha with *Pitta* preponderance, leading to Vata and Pitta *Prakopa* (vitiation) upon nail damage, thus causing pain and discomfort. *Kunakha* is also known as *Chippa* or *Anguliveshtaka* in ancient Ayurvedic literature, with Acharya Charaka referring to it as *Aksata*. This case report demonstrates significant relief from paronychia through comprehensive Ayurvedic treatment, including *Shaman Chikitsa* (pacifying treatment).

**Case presentation:**

A south 63-year-old Asian male patient came to Mahatma Gandhi Ayurved College Hospital and Research Center Salod (Hirapur), Wardha, Maharashtra, India. Patient had symptoms of pain and blackish discoloration in index finger nail and thumb finger nail of right hand since 3 months. Associated with pain patient has swelling and tenderness along with pus discharge which was on and off in origin.

**Conclusion:**

The findings highlight the potential efficacy of Ayurvedic interventions in managing chronic and recurrent nail infections, offering a promising alternative for conditions that are otherwise challenging to treat with modern medicine. Despite being resistant to conventional modern therapies, the patient experienced substantial improvement, with no recurrence observed even after 1 year post-treatment.

## Introduction

All nail infections are referred to as *Kunakha* in Ayurveda. Ku means “bad,” and *Nakha* means “nail” in Hindi. Following the administration of external and palliative treatment, a single case study demonstrating *Kunakha* condition, which had shown resistant to therapy in modern science, produced optimistic outcomes (*Shaman Chikitsa*) [1].

A bacterial or fungal infection of the hand or foot that affects the area where the nail and skin touch at the side or the base of a fingernail is known as paronychia. It typically affects housewives and housemaids who frequently engage in water-related work. It is a relatively straightforward illness that often improves gradually over the course of a week, but the issue emerges when pus continues to form and worsens after exposure to water, chemicals, or unclean substances [2]. According to universal consensus, the ancient science of cosmetology known as Ayurveda’s Kshudraroga *Kunakha* is associated to paronychia. One of the most prevalent physical symptoms of nail illness is *kunakha* (nail discolouration) [3]. *Kunakha* is associated to *Asthi dusthi* (bone tissue), a condition characterised by a vitiated *Tridosha* with *Pitta* preponderance. *Mala* (by-product) of *Asthi dhatu* is *Nakha* (nail). *Asthi dushti* is brought on by the disease’s chronicity [4]. In concern with cosmetic view, this clinical condition affects the appearance of hands, is embarrassing, even disfiguring, and hampering person’s day‑to‑day activity. It induces severe ugliness to hands due to chronic recurrent infection [5]. This illness has been identified as the disease *Chippa*. The *Kunakha* condition, which is caused by a slight rise in *Doshas*, is the same one. The 13th chapter of *Nidana Sthana*, which explains the *Nidana* (causes) and *Lakshana* (signs) of *Kshudra Rogas* (minor ailments), among them *Chippa* and *Kunakha*, refers to *Kunakha* as one of the *Kshudra Rogas*. Any damage to the nail results in *Vata* and *Pitta Prakopa*, which in turn causes pain [6]. Another name for the *Cippa Roga* is the *Anguliveshtaka* (covering to nail). This condition was referred to as *Aksata* by Acharya Charaka [7].

In this case report, we got significant relief in paronychia with complete Ayurveda treatment. The relapsing nature of paronychia is seemed to be stopped even after 1 year of stopping treatment in this case.

## Case presentation

### Patient information

A 63-year-old male patient came to Mahatma Gandhi Ayurved College Hospital and Research Center Salod (Hirapur), Wardha. Patient had symptoms of pain and blackish discoloration in index finger nail and thumb finger nail of right hand since 3 months. Associated with pain patient has swelling and tenderness along with pus discharge which was on and off in origin.

### Family history

- Mother—no history of illness
- Father—no significant history
- Siblings—no significant history

### Past history

Patient had similar complaints before 1 year.

No History of DM, TB, or asthma.

Known case of hypertension since 2 years.

### Personal history

Patient has addiction to tea, tobacco, betel nuts, and occasional alcohol.

- Diet—mixed diet (both vegetarian and nonvegetarian)
- Sleep—disturbed sleep, sleeping hours are not fixed.
- Bowel—not satisfactory, goes 1–2 times for defecation.

## Clinical findings

**Fig. 1:**
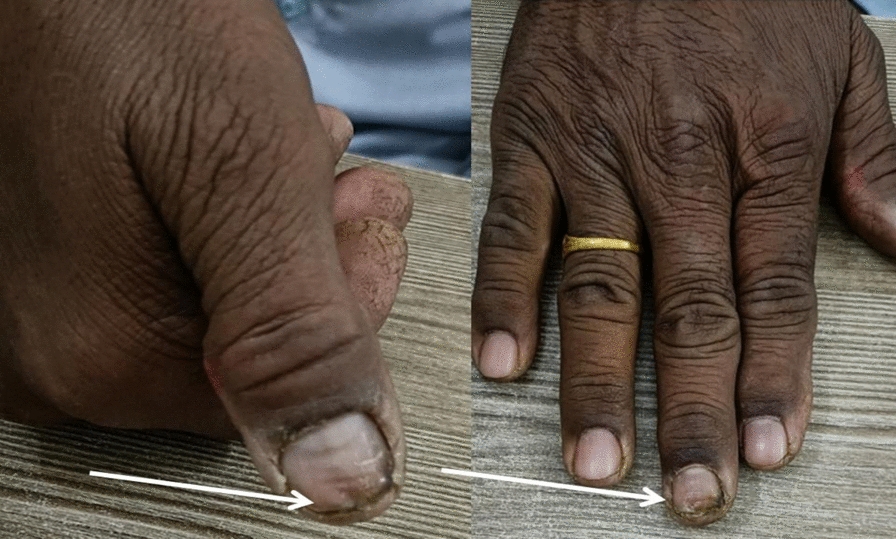
Paronychia (inflammation and discoloration; indicated by white arrow) of the thumb on the right hand (**A**) and middle digit (**B**) before treatment

A patient has edema, erythema, and severe throbbing pain along with nail fold. Nails show blackish discoloration (Fig. 1). Tenderness was present.

### General examination

When the patient came to OPD, he was thoroughly examined and complete history was taken. Patient was supportive, oriented to time and place.

Vitals—Temperature—97.3, Pulse—84/min, Respiratory rate—18/min, Blood pressure—130/90 mmHg.

### ***Asthavidhaparisha*** (eight vitals according to ayurveda) [8]

- *Nadi* (pulse)—66/min
- *Mutra* (urine)—3 to 4 times/day
- *Mala* (feces)—3 to 4 times/day, satisfactory
- *Jivha* (tongue)—Sama (coated)
- *Shabda* (speech)—Spashta (normal)
- *Sparsha* (touch)—Anushnashita (normal)
- *Drik* (vision)—no pallor (normal)
- *Akruti* (shape)—Madhyam

## Timeline

See Table 1 and Fig. 1.

**Table 1:** Timeline

15/10 /2021	Patient noticed discoloration of nails with pus discharge, pain at nails of right hand
18/10/2021	He saw a general practitioner in this regard and took medication for roughly 15 days, during which time his symptoms improved
20/11/2021	He started same signs and symptoms and consulted same physician and took medications for 15 days
10/12/2021 – 5/1/2022	He had done 4–5 visits for the same regard and the condition was relapsing
14/1/2022 to 1/3/2022	He came to OPD of Mahatma Gandhi Ayurved College Hospital and Research Center Salod (Hirapur), Wardha, Maharashtra, India and complete Ayurved treatment is started and successful result is obtained

## Diagnostic assessment

Paronychia is typically diagnosed with a physical exam. An X-ray may occasionally be used to check for a foreign body or any signs of osteomyelitis, a bone infection that can develop when a chronic fungal infection causes paronychia. But in this case we have not done X ray as the patient was from economically poor background.

### Pain scale (VAS)

**Fig. 2:**
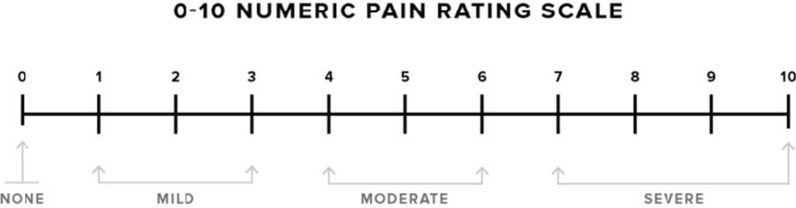
Pain scale (Hayes and Patterson, VAS scale, 10 Oct 2019, https://www.healthline.com/health/pain-scale) [9]

Pain scale is used for assessing pain of this patient, 0—none, 1 to 3—mild, 4 to 6—moderate, 7 to 10—severe (Fig. 2).

### Grading of tenderness [10]

- 0—no tenderness
- 1—mild tenderness, without wincing
- 2—tenderness with grimace
- 3—jump sign positive
- 4—jump sign with noxious stimulus.

## Therapeutic intervention

See Table 2.

**Table 2:** Therapeutic intervention

Type of treatment	Drug name	Dose	Administration time	Duration	Anupana
Internal	*Gandhak rasayan*	250 mg (BD)	After meal	15 days	Lukewarm water
	*Praval panchamrit*	250 mg (BD)	After meal	15 days	Lukewarm water
	*Triphala gugulu*	250 mg (BD)	After meal	15 days	Lukewarm water
External	*Karanja tail*	–	Two times a day	15 days	–

## Follow up and outcomes

Adverse and unanticipated events—no adverse drug reaction or any adverse drug effect is encountered during the clinical study.

## Discussion

**Fig. 3:**
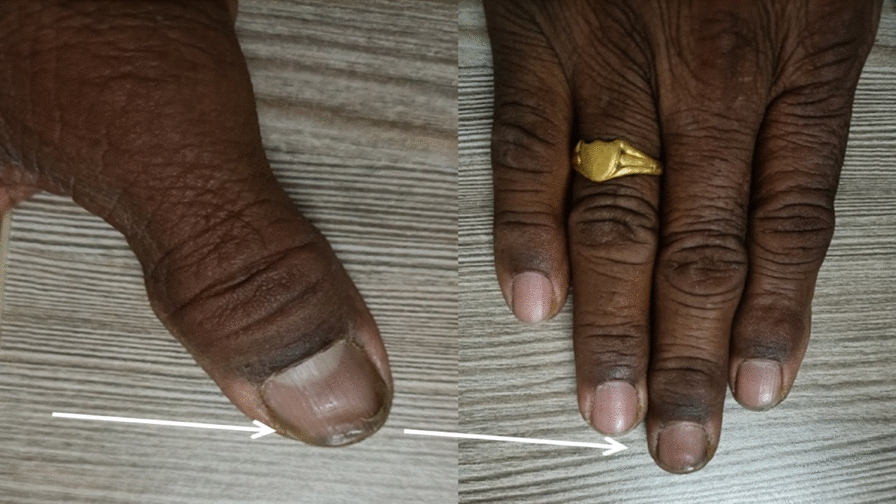
Normal appearance of thumb on right hand (**A**) and middle digit (**B**) after treatment (white arrow)

In Ayurveda, the *Kshudraroga Kunakha* is usually connected to paronychia. *Kunakha* is associated with *Asthi dusthi*, an illness characterized by vitiation of *Tridosha* with a predominance of Pitta. *Mala* of *Asthi dhatu* is *Nakha*, or nail. *Asthi dushti* is caused by the chronicity of the condition. In the twentieth chapter of *Chikitsa Sthana*, Acharya Sushruta provided an explanation for *Kunakha* management. This ailment, which Acharya Charaka named *Aksata*, is brought on by vitiated *Rakta* (blood tissue) and *Mamsa* (muscle tissue) and is characterized by severe *Daha* (burning) and *Paka* (inflammation) under the nails [11]. In this case we got significant result in swelling, inflammation (Fig. 3) and Pain on VAS scale (Fig. 2, Table 3). This patient had taken medication at our OPD as per timeline given above (Table 1).

**Table 3:** Follow up and outcomes

Criteria	BT	First follow up	Second follow up	Third follow up	AT
Pain	7	6	4	3	0
Tenderness	3	2	1	1	0
Difficulty in grasping things	Pain on grasping things	Moderate pain on grasping things	Mild pain on grasping things	Mild pain on grasping things	No pain on grasping things

Pain, discoloration, brittleness, and other symptoms in this patient indicate that the vitiated Doshas are *Pitta* and *Vata*, which in turn indicate *Rakta Dhatu* and *Asthi Dhatu*. Treatment for *Vata*-*Pitta Shaman*, *Shothahara* (anti-inflammatory), and *Raktaprasadan* (nourishes blood) was developed and carried out after taking into account all relevant aspects and disease situations [12]. Gandhak Rasayan has a number of qualities, including antiviral, antibacterial, anti-inflammatory, and anti-pruritic. Action: Gandhak Rasayan’s antibacterial activity may help reduce the signs and symptoms of guttate psoriasis; likewise, its anti-pruritic action relieves itching. According to Ayurveda, Gandhak Rasayan, which induces sweating and helps maintain the ideal state of *Rakta Dhatu* (blood), also functions as a rejuvenator by eliminating Pitta and *Kaphadosha* (humours) from the body and relieving itching owing to its anti-pruritic property [11]. Triphala Guggulu is directly indicated in *Shotha* (inflammation), wherein its anti-inflammatory action is aided by *Pippali* (Piper longum Linn.), *Triphala* (Terminalia chebula Retz., Terminalia bellerica Gaertn. Roxb., and Emblica officinalis Gaertn.), and *Guggulu* (Commiphora wightii Arnott Bhandari). Its components have extremely strong anti-microbial properties, which makes it the recommended medication for inflammatory and infectious disorders [13]. *Praval Panchamrit* is added as it works on *Asthi dhatu* and provides nourishment to *Asthi* and in turn makes healthy nails which are the mala of *Asthi dhatu* [14]. For local application, *Karanj oil* is used. *Karanj oil* is used for its *Kandughna* (anti-pruritic) property. It includes *Langali* (Gloriosa superba), *Saptacchada* (Alstonia scholaris), *Karanj* (Pongamia pinnata), and *Arka* (Calatropis procera).

*Vatsanabh* (Aconitum ferox), *Gomutra* (Cowurine), *Brungaraj* (Eclipta alba), and *Chitraka* (Plumbago zeylanica). It is frequently used for a number of skin conditions. Karanj oil has therapeutic qualities and is used to treat skin conditions such as abscesses and itchiness [8]. Table 2 (Treatment).

## Conclusion

*Kunakha* (Paronychia) can be easily treated with Ayurvedic line of treatment. As Nails are the by-product of *Asthi dhatu*, working on *Asthi dhatu* could give significant results. In this case, we got significant result with *Gandhak rasayan, Praval panchamrit, Triphala gugulu* and *Karanja oil* in 15 days. For the purpose of verifying the medications, more study can be conducted using the same technique on a series of instances. Additionally, other nail conditions with similar symptoms can also benefit from treatment. This is a minor illness and people use antibiotics for such ailments which can cause antibiotic resistance. This poly-herbo-mineral can help to get good results in Paronychia without any relapses.

## Data Availability

Not applicable.

## References

[1] Thulasi TV. Effect of jalukavacharana in nail bed infection - a single case study. J Ayurveda Integr Med Sci. 2022;7(1):411–5.

[2] Rigopoulos D, Larios G, Gregoriou S, Alevizos A. Acute and chronic paronychia. Am Fam Phys. 2008;77(3):339–46. 18297959

[3] Victoria Devi L, Khagen B. Contribution of Ayurvedic classics on Kshudra roga with special reference to cosmetic diseases. Int J Ayur Pharm Res. 2016;4:83–7.

[4] Sawarkar P, Sawarkar G. Contribution of Ayurveda for management of paronychia: a case report. J Indian Syst Med. 2019;7:240–4.

[5] Relhan V, Goel K, Bansal S, Garg VK. Management of chronic paronychia. Indian J Dermatol. 2014;59:15–20. 24470654 10.4103/0019-5154.123482 PMC3884921

[6] Susruta Samhita, Nibandhasangraha commentary of Dalhana Acharya. nyayachandrika of Gayadasa In: Hindi translation by Krishna Takral, 2ndpartsarir,chikitsa and Kalpa stana. Kshudraroga chikitsitam. Varanasi: Chowkhamba orientalia; Reprint 2014; p. 394.

[7] Agnivesa. Caraka Samhita revised by Caraka and Drdhabala with the Ayurveda Dipika Commentary of Chakrapanidatta. In: Vaidya Jadavji, Trikamji Acharya (Editor). Svayathu chikitsitham. Varanasi: Chowkhamba Krishnadas Academy; Reprint 2015; p. 490.

[8] Biswas A, Deshpande AM, Deshpande MA. An Ayurvedic approach to Vipadika (palmoplantar psoriasis): a case study. J Pharm Res Int. 2021;24:267–73.

[9] Jadhav D, Deshpande MA, Deshpande AM, Urkude M. Management of Parshnishool (Heel Pain) Due to Vatakantaka (Calcaneal Spur) with Agnikarma (Therapeutic Burn).—a case report. Int J Life Sci Pharma Res. 2023;13(5): L358–63.

[10] Kannan P. Management of myofascial pain of upper trapezius: a three group comparison study. Global J Health Sci. 2012;4(5):46. 10.5539/gjhs.v4n5p46 PMC4776923 22980377

[11] Chhabra DA, Kuchewar DV, Joshi DT. Avascular necrosis through ayurveda perspective-a case report. Int J Life Sci Pharma Res. 2023;13(1):L218-223.

[12] Sachin PK, Dattatray PV, Madhav PS. Role of gandhak rasayan in shushka vicharchika (eczema). Ayushdhara. 2016;4(3):1207–10.

[13] Dwivedi AK, Tiwari A, Gupta A. Management of sandhishula and sandhishotha-an ayurvedic approach.

[14] Sharma U, Rani S. Review on Praval Panchamrita Rasa: a natural calcium supplement. Sch Int J Tradit Complement Med. 2023;6(9):134–9.

